# Functional assessment of brain development in fetuses that subsequently deliver very preterm: An MRI pilot study

**DOI:** 10.1002/pd.6498

**Published:** 2023-12-21

**Authors:** Lisa Story, Alena Uus, Megan Hall, Kelly Payette, Spyros Bakalis, Tomoki Arichi, Andrew Shennan, Mary Rutherford, Jana Hutter

**Affiliations:** ^1^ Department of Women's and Children's Health King's College London St Thomas' Hospital London London UK; ^2^ Centre for the Developing Brain School of Biomedical Engineering and Imaging Sciences King's College London St Thomas' Hospital London London UK; ^3^ Fetal Medicine Unit St Thomas' Hospital London London UK; ^4^ Radiological Institute University Hospital Erlangen Erlangen Germany

## Abstract

**Objectives:**

To evaluate changes occurring in the fetal brain prior to very preterm delivery using MRI T2* relaxometry, an indirect assessment of tissue perfusion.

**Method:**

Fetuses that subsequently delivered spontaneously <32 weeks gestation and a control cohort were identified from pre‐existing datasets. Participants had undergone a 3T MRI assessment including T2* relaxometry of the fetal brain using a 2D multi‐slice gradient echo single shot echo planar imaging sequence. T2* maps were generated, supratentorial brain tissue was manually segmented and mean T2* values were generated. Groups were compared using quadratic regression.

**Results:**

Twenty five fetuses that subsequently delivered <32 weeks and 67 that delivered at term were included. Mean gestation at MRI was 24.5 weeks (SD 3.3) and 25.4 weeks (SD 3.1) and gestation at delivery 25.5 weeks (SD 3.4) and 39.7 weeks (SD 1.2) in the preterm and term cohorts respectively. Brain mean T2* values were significantly lower in fetuses that subsequently delivered before 32 weeks gestation (*p* < 0.001).

**Conclusion:**

Alterations in brain maturation appear to occur prior to preterm delivery. Further work is required to explore these associations, but these findings suggest a potential window for therapeutic neuroprotective agents in fetuses at high risk of preterm delivery in the future.

## Key points

What is already known on this topic?

- Few studies have been conducted exploring brain development prior to preterm birth; however, a reduction in the cerebral cortex and extracerebrospinal fluid volumes and alterations in functional connectivity have been reported in fetuses that subsequently deliver preterm.

What does this study add to what is known?

- Mean T2* relaxometry values are significantly lower in fetuses that subsequently deliver <32 weeks in comparison with fetuses that deliver at term, indicating that alterations in tissue perfusion may be occurring prior to preterm birth.

## 1 INTRODUCTION

Very preterm birth, less than 32 weeks gestation, occurs in 1.3% of pregnancies in England and Wales every year^1^ and is associated with significant mortality and morbidity. The incidence of neurological complications is high, including a 36% incidence of intraventricular haemorrhage (IVH)^2^ and 27% risk of periventricular leukomalacia.^3^ One in five of these children are subsequently diagnosed with motor or cognitive developmental delay at 2 years of age, with approximately 7% receiving a diagnosis of cerebral palsy.^4^ Neuropsychiatric and behavioural disorders are also more prevalent in later life, with a five‐fold increased risk of attention deficit hyperactivity disorder and double the risk of anxiety disorders.^5^

The pathogenesis of brain injury associated with spontaneous preterm birth (sPTB) has not been fully elucidated but is likely to be multifactorial. The two main mechanisms are hypothesised to be ischaemia and inflammation, which may be concomitant and cumulative, insults that are more prevalent and potent in the immature brain.^6^ Inflammation may arise from postnatally acquired sepsis; however, there may also be antenatal antecedents.^6^, ^7^ Inflammation/infection has been implicated in the aetiology of sPTB itself.^8^ It is also well documented that the presence of chorioamnionitis diagnosed on placental histology following delivery is associated with a further significant increase in the risk of adverse neonatal neurological sequelae.^9^

Until recently, tools to assess in utero changes in brain development prior to sPTB have been limited. MRI is a non‐invasive imaging modality routinely used in obstetric practice for anatomical assessment of the fetal brain and body.^10^ We have previously demonstrated using motion corrected anatomical fetal MRI that cerebral cortical volumes and extracerebral spinal fluid volumes are reduced in fetuses that subsequently deliver preterm,^11^ supporting the hypothesis that alterations in brain development may precede sPTB. However, the ability of MRI to provide functional information about fetal tissue^12^ is now possible due to novel acquisition and processing techniques.^13^, ^14^ T2* imaging harnesses the fact that oxygenated and deoxygenated haemoglobin have different paramagnetic properties, thereby providing an indirect assessment of tissue oxygenation and metabolic activity in the fetal brain.

This study aims to assess whether alterations in mean brain T2*values, as a proxy marker for tissue oxygenation, occur in the fetal brain prior to very preterm birth.

## 2 METHODS

A case control study was performed, utilising datasets acquired prospectively as part of four UK NHS Research Ethics Committee (REC) approved studies (Placental Imaging Project REC 16/LO/1573 2016, MRI assessment of in utero lung development in fetuses that deliver less than 32 weeks gestation REC 19/LO/0736 2019, Antenatal assessment of fetal infection utilising advanced MRI Protocols REC 19/SS/0032 2019, and Individualised risk prediction of adverse neonatal outcome in pregnancies that deliver preterm using advanced MRI techniques and machine learning REC 21/SS/0082 2021). All datasets were collected between 2016 and 2023 at St Thomas' Hospital London. Written consent was acquired from all participants prior to commencing participation.

Women at high risk of sPTB had been prospectively recruited if they had:

1) Bulging membranes <32 weeks gestation;
2) Preterm prelabour rupture of membranes <32 weeks gestation confirmed on clinical speculum examination.
3) A >50% risk of preterm delivery as determined by the QUIPP app,^15^ a validated algorithm incorporating previous medical and obstetric history, ultrasound‐derived cervical length and quantitative fetal fibronectin.^15^

A control group was selected from the same studies as the high‐risk group. These women were imaged at gestations similar to the preterm cohort and had uncomplicated pregnancies that subsequently delivered at term. Demographic data including maternal age, body mass index and ethnicity were collected from medical records.

For both groups, inclusion criteria included 18–32 weeks gestation at the time of MR imaging, singleton pregnancy, ability to give informed consent, not in active labour, complete imaging of the fetal brain (the focus of some of the studies had been the placenta) without major imaging artefacts on the T2* imaging sequences such as poor signal to noise ratio. Exclusion criteria included known fetal chromosomal or structural abnormalities, active labour, claustrophobia or a recently sited metallic implant, maternal medical conditions, or pregnancy complications such as pre‐eclampsia, fetal growth restriction and gestational diabetes.

Written consent was obtained from all participants and an MRI scan was performed on a 3T imaging system (Phillips Achieva). Women were scanned in a supine position using a 32‐channel cardiac coil. The total imaging time was approximately 1 hour with monitoring of maternal heart rate, pulse oximetry and blood pressure. A break was offered to all participants halfway through the scan. Previous optimisation of sequences had been performed to ensure that acoustic noise during acquisition was below 98 db. Localiser preparation scans were first performed followed by anatomical scans of the whole uterus using 2‐D Turbo Spin Echo sequences in five orientations encompassing the fetal brain.^16^ A B0 map was acquired and manual shimming using an in‐house pipeline was performed. For T2* mapping, a 2‐D multislice multi echo gradient echo planar imaging sequence with four echo times was acquired in the coronal orientation to the mother covering the entire uterus wherever possible. Parameters included: field of view = 360 mm (320–400 mm) × (60–120 mm), resolution 3 mm isotropic, repetition time = 2.6 s, echo times between 11.6 and 182 ms, 1–2 dynamics.

**FIGURE 1:**
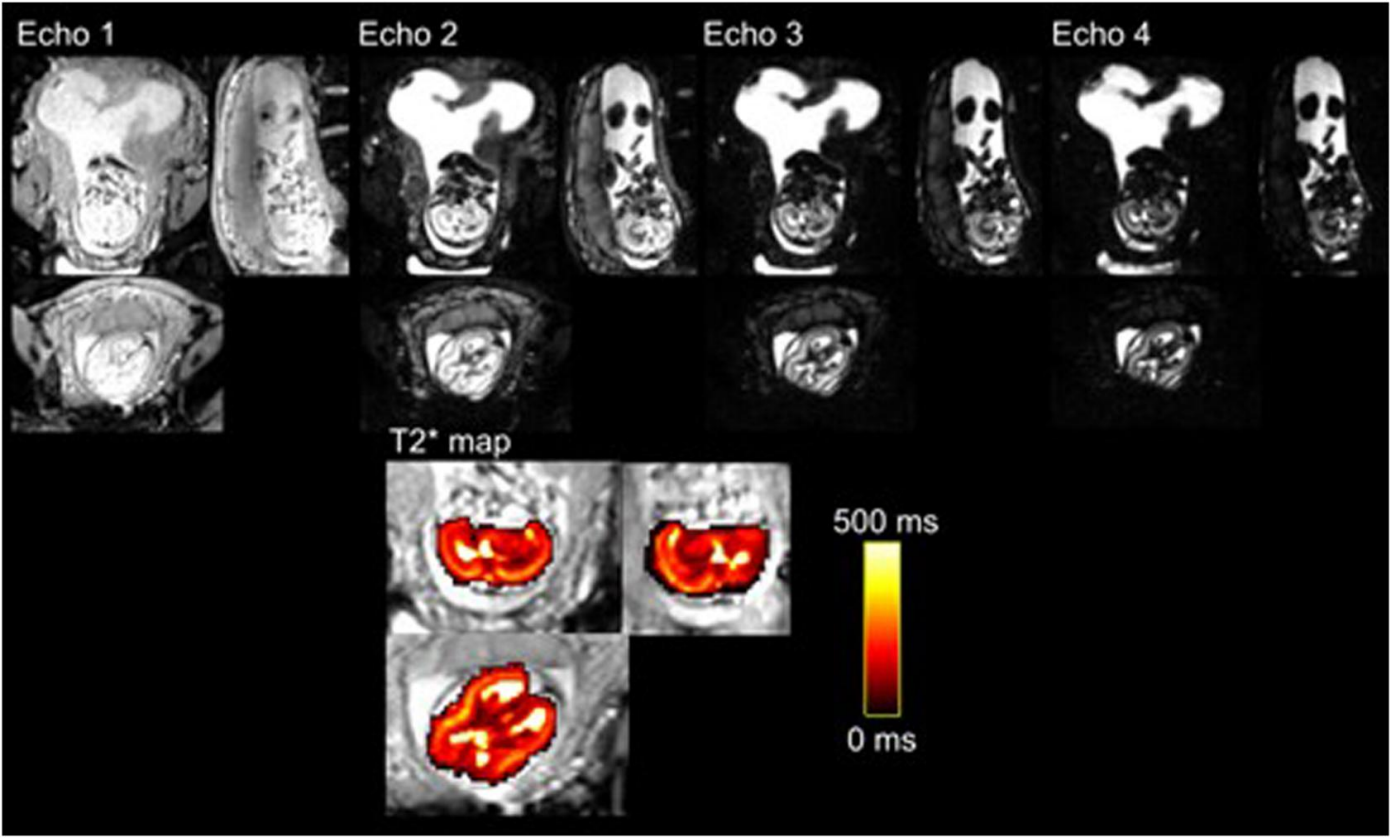
Exemplary dataset from the studied control cohort, the top row individual echo times, bottom row resulting T2* map in the brain overlaid over the data from the first echo time.

All scans were reported by a perinatal radiologist for overt pathology. The total supratentorial brain tissue was manually segmented, with the exclusion of the extra cerebral cerebrospinal fluid (eCSF), from T2* maps by one experienced observer (LS, 15 years' of experience in fetal MRI). Good inter observer variability had previously been confirmed by a second observer (JH, 9 years' experience of fetal MRI) with an intraclass correlation co‐efficient >0.99. A conservative margin of segmentation was used to avoid non‐cerebral tissue (see Figure 1) and thresholding was applied to avoid all cerebrospinal fluid. Mono‐exponential decay models were fitted to the data acquired for T2* maps using an in‐house python script to obtain proton density and T2* maps: 10 random initialisations were performed and the voxel‐wise median value was used. Additional histogram‐based evaluation was performed for the T2* values and skewness values were obtained as a descriptive measure.

Pregnancy outcome data regarding gestation at delivery, sex of infant and any maternal or neonatal complications until discharge from hospital were collected from all participants from clinical records. Details of any clinically indicated antenatal ultrasound scans within 1 week of MRI imaging were also collected as well as details of any intracranial abnormalities identified on postnatal imaging. Where available, details of placental histology were recorded. All placentas were reported by a specialist perinatal pathologist in accordance with the Amsterdam Criteria.^17^

## 3 STATISTICAL ANALYSIS

Statistical analysis was performed using SPSS (IBM) version 28.0. Data were first checked for normality. Where a normal distribution was not present, data were transformed. The student *t*‐test was used to compare continuous demographic data between groups, and Chi squared where data was categorical. Quadratic regression was used to assess the relationships between mean T2* values between the control group and the preterm cohort accounting for the effects of gestation and ethnicity and linear regression was used to assess differences in supratentorial brain volumes and skewness. No formal power calculations were performed as this was a pilot study exploring a novel hypothesis.

## 4 RESULTS

Data from 25 women with complete imaging of the fetal brain who subsequently delivered <32 weeks of gestation were included. Fourteen of these women had ruptured membranes at the time of imaging. Ten additional datasets were available from women who were recruited as being at high risk of preterm birth, but seven of these women delivered after 32 weeks gestation and three developed concomitant diabetes and were excluded.

Data sets from 67 low‐risk pregnancies who delivered >37 weeks gestation and had complete imaging of the fetal brain at similar gestations were selected as the control group.

The demographic and delivery parameters of both groups can be seen in Table 1.

**TABLE 1:** Demographic and delivery parameters of participants.

Parameter	Preterm group (*n* = 25)	Control group (*n* = 67)	*p* Value
Maternal age (years)			
Mean (SD)	32.2 (5.4)	33.9 (4.1)	0.1
Range	21.5–41.8	22.1–45.1	
BMI			
Mean (SD)	24.1 (3.3)	23.3 (3.0)	0.9
Range	18.3–30.8	18.2–32.5	
Ethnicity (%)			
White	52	91	**0.01**
Mixed	8	3	
Asian	20	3	
Black	16	3	
Other	4	0	
Parity (%)			
0	60	61	0.4
1	32	31	
2	4	8	
3	4	0	
>3	0	0	
Gestation at MRI (weeks)			
Mean (SD)	24.5 (3.3)	25.4 (3.1)	0.4
Range	19.3–29.7	19.3–31.7	
Gestation at delivery (weeks)			
Mean (SD)	25.5 (3.4)	39.7 (1.2)	
Range	20.1–30.3	37–42.1	
Outcome (%)			
Live to discharge	64	100	
Intrapartum death	24	0	
Neonatal death	12	0	
Sex of infants (%)			
Male	48	49.2	0.18
Female	40	50.8	
Undetermined^a^	12	0	
Neonatal complications (%)	*N* = 19	*N* = 67	
Intraventricular haemorrhage			
Grade 3/4	5	0	
Respiratory distress syndrome	84	0	
Bronchopulmonary dysplasia	21	0	
Necrotising enterocolitis	16	0	

Abbreviation: BMI, body mass index.

^a^ Unable to determine because of previability.

**FIGURE 2:**
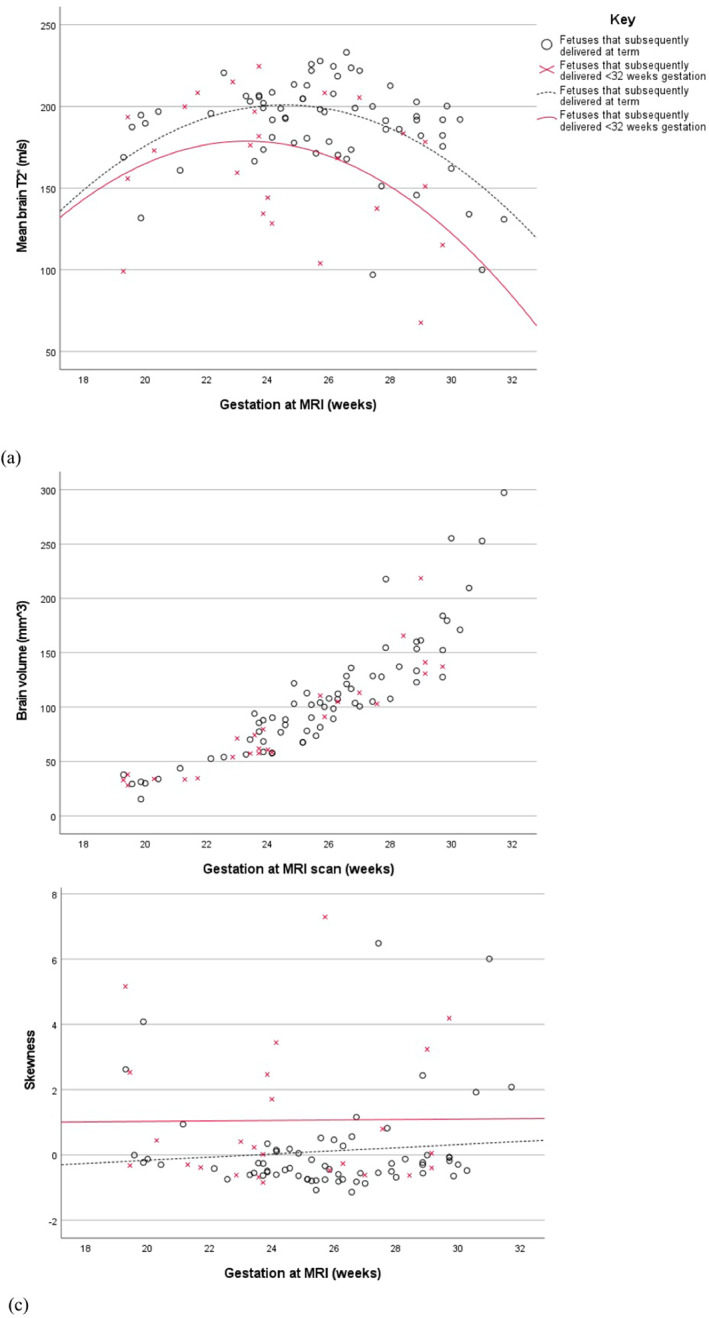
Graphs illustrating (A) mean brain T2* values (B) total brain volume and (C) histogram skewness in fetuses that subsequently delivered <32 weeks gestation and those that delivered at term.

No overt cerebral pathology was identified on fetal MRI in any of the participants. Mean T2* values were significantly lower in the fetuses that subsequently delivered preterm (*p* < 0.001) (Figure 2). Ethnicity did not appear to impact this finding (*p* = 0.681). Although numbers were small in the specific subgroups, this finding persisted in both cases with (*p* = 0.025) and without (*p* = 0.004) ruptured membranes at the time of imaging. There was no difference in total brain volume between the two groups (*p* = 0.51).

Brain T2* skewness (*p* = 0.014) was significantly higher in the preterm cohort in comparison to the control group.

Of the preterm born infants, six had Doppler blood flow assessments of the middle cerebral artery (MCA) as part of clinical ultrasound scans within 7 days of MRI imaging. No fetuses had an estimated fetal weight <10^th^ centile but the MCA Doppler (MCA) was below the 5^th^ centile in 3/6 of these cases.

**FIGURE 3:**
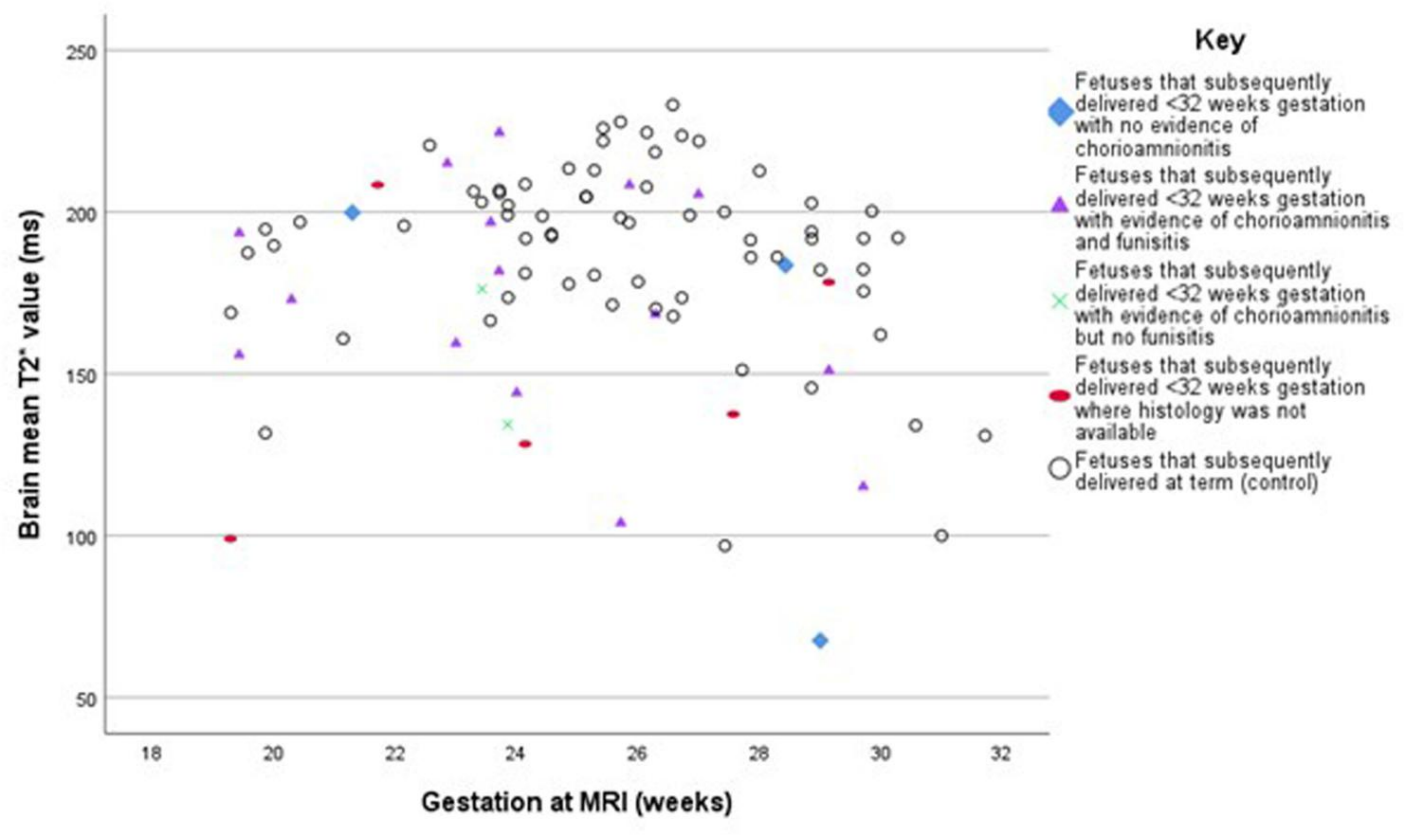
Brain mean T2* values in fetuses that delivered preterm with and without a histological diagnosis of chorioamnionitis +/− funisitis.

Placental histology was available in 21 of the preterm cases, with 17 showing evidence of chorioamnionitis. Of these, 15 additionally showed evidence of funisitis. Subgroup analysis was not possible due to the limited numbers without chorioamnionitis, but Figure 3 illustrates the impact of chorioamnionitis on brain mean T2* values.

As only one neonate had a subsequent significant brain injury, grade IV IVH, numbers were not sufficient to correlate the postnatal outcome with mean fetal brain T2* values.

## 5 DISCUSSION

This study has demonstrated that mean cerebral T2* values were significantly lower in fetuses that subsequently delivered very preterm. No overt pathology was identified on clinical review of the images and no differences were identified in supratentorial brain volume between the two groups.

Although mean T2* values have been assessed in other fetal organs^18^ including the liver^19^, spleen^20^ and lungs,^12^, ^20^ few studies have previously assessed fetal brain development using this technique.^21^, ^22^, ^23^ Previous work has assessed the fetal brain using T2* relaxometry in a group of healthy pregnancies as well as those complicated by placental insufficiency on a 3T MRI scanner between 21^+5^ and 38^+4^ weeks gestation.^23^ In agreement with the findings in this study, values decreased between 21^+5^ and 38^+4^ weeks of gestation in uncomplicated pregnancies in a linear manner.^23^ Blazejewska et al. characterised T2* values in the grey matter of the fetal brain between 20 and 36 weeks gestation.^21^ They noted that T2* values were approximately twice those found in adults and decreased with increasing gestational age for 20 weeks. This overall trend is consistent with the decrease in mean T2* values derived from all supratentorial tissues observed in our study between 22 and 32 weeks of gestation. Vasylechko et al. assessed cerebral T2* values in three regions, the frontal white matter, thalamic grey matter and occipital white matter, and also demonstrated a linear decrease in values between 22 and 38 weeks of gestation in 23 fetuses.^22^ However, all examinations were undertaken for clinical indications including ventriculomegaly, transposition of the great arteries, and maternal toxoplasmosis, and only three had a structurally normal appearance to the brain.^22^ The studies by both Blazejewska and Vasylechko used 1.5T imaging systems,^21^, ^22^ whereas the current study was undertaken at 3T. While the general decrease with increasing field strength of T2* has been described extensively, the higher T2* on low field increases the dynamic range available, particularly of interest for the typically low T2* observed in pathological cases and in late gestation.^24^, ^25^ To the best of our knowledge, this is the first study to report on control fetuses below 20 weeks gestation, finding an increase in mean brain T2* values between 19.3 and 22 weeks. The increase in activity is implicit with the increase in cell density during this period.^26^

To our knowledge, no previous studies have utilised mean T2* values to characterise brain development in fetuses that subsequently deliver very preterm. However, work by Thomason et al. has demonstrated alterations in functional connectivity in fetuses that were subsequently delivered prior to 37 weeks in a left hemisphere pre‐language region. Thirty‐six fetuses were included, 14 of which delivered preterm.^27^ Although numbers in both studies are currently small, they do indicate that changes in brain development occur prior to preterm delivery, which may have implications for early brain function and may predispose them to acquired injury peripartum.

Alterations in neuronal morphology and angiogenesis associated with prematurity may in part explain the lower mean T2* values observed in this study in the preterm cohort. The aetiology of sPTB is complex and likely multi‐factorial but infection/inflammation is thought to play a significant role, particularly at early gestations.^28^ In keeping with this, 81% of placentas analysed from the preterm cohort in this study had histopathological evidence of chorioamnionitis post‐delivery, with 88% of these having evidence of funisitis indicating a fetal inflammatory response.^11^ Inflammatory cytokines associated with the onset of sPTB itself are also thought to further contribute to neonatal brain injury such as IVH and white matter injury including punctate lesions and periventricular leukomalacia.^29^ Whilst there were no signs of overt injury on the fetal MRIs, our findings support theories that injurious changes may begin in utero. We have previously demonstrated that although no difference in supratentorial brain volumes between fetuses that subsequently deliver preterm compared to those that deliver at term was detected, significantly lower eCSF and cortical volumes were observed.^11^

On a microstructural level, abnormal neuronal growth and morphology with a reduction of dendritic growth has been reported in a mouse model of lipopolysaccharide induced preterm birth (LPS).^30^ These alterations in developmental processes may represent altered metabolic requirements of tissue during these developmental processes, consequently reflected in a reduction in mean cerebral T2* values in the preterm cohort in comparison with the control group. Reductions in vascular density, pericyte and astrocyte coverage of the blood vessels and astrogliosis in the cerebral cortex and white matter have also been observed in a LPS‐induced ovine model of preterm birth.^31^ The consequences of this may be a reduction of cerebral perfusion also contributing to the lower mean T2* values observed in this study.

A reduction in global cerebral perfusion has also been demonstrated in the preterm born neonates. Preterm born infants at term equivalent age were observed to have both global and regional reductions in cerebral perfusion compared with those born at term, using 3T arterial spin labelling MRI.^32^ Relative alterations in regional perfusion, standardised to global perfusion, were also assessed, demonstrating a proportional reduction to the sensory cortex, the anterior cingulate cortex and insula in the preterm born cohort.^32^ Although multiple confounding factors occur in the neonatal period, our findings of a reduction in mean T2* values observed in fetuses that subsequently deliver preterm, may represent antenatal antecedents of neonatal alterations in cerebral perfusion.

Cerebral blood flow has been indirectly assessed by two groups in fetuses that subsequently deliver preterm using Doppler ultrasound assessment of the MCA Pulsatility Index (PI).^33^, ^34^ Both reported lower MCA PI values in fetuses that were delivered prematurely. Although such findings are often attributed to an increase in cerebral blood flow, it should be noted that the MCA PI is a measure of flow impedance and represents an indirect assessment of blood flow only. In addition, it represents only one component of the cerebral vasculature. The neonatal findings of Bouyssi–Kobar indicate that flow to different regions of the brain is also altered to varying degrees in preterm born neonates^32^ and the study by Disidier et al. indicate that alterations may be on a microvascular level.^31^

The characteristics of the T2* values can be explored further using measures of skewness. This assesses the degree of symmetry of the frequency distribution. Our findings suggest that skewness is significantly higher in the brains of fetuses that are later born preterm compared with the control group and there is more heterogeneity in the T2* values. This may be attributable to the variable time between the MRI scan and delivery and in the group where the membranes had been ruptured, the time between this event and the MRI scan. If infection is in part attributable to the differences observed between groups, then this may also represent different stages of the process.

Lower mean brain T2* values identified in the preterm cohort may represent alterations in tissue perfusion/metabolism occurring in fetuses prior to sPTB. This is of potential significance in the future as neuroprotective agents such as magnesium sulphate are currently advocated only when women are at imminent risk of preterm labour.^35^ However, if alterations in brain physiology occur days or weeks before delivery itself, in the future this period could be considered as a candidate neuroprotective agent in the future. If these findings predate irreversible damage, they could also be used to determine the optimal timing of delivery.

### 5.1 Strengths and limitations

To our knowledge, this is the first study to assess the functional properties of cerebral tissue in fetuses that subsequently deliver preterm. We have demonstrated good reproducibility of this technique and our findings provide further evidence that alterations in brain development may occur in the days and weeks preceding sPTB.

It should be noted that the mean T2* values provide only an indirect reflection of tissue perfusion/metabolism. At present, only total supratentorial brain tissue has been assessed and differences may occur within different regions of the brain. Previous work by Vasylechko has indicated that mean T2* values were higher in the white matter than deep grey matter in fetuses with uncomplicated pregnancies,^22^ indicating that regional differences do occur.

It is also important to highlight that currently there remains a significant disparity in the ethnicity of women between the preterm group and control groups; however, we did try to account for this to some extent within the statistical analysis. This remains an important consideration and is a well‐recognised issue within maternity research.^36^ Efforts are currently underway to explore the barriers to research participation in women with healthy pregnancies from different backgrounds, as this will be essential to address in future studies.

Further work is required to explore the association between fetal mean brain T2* values and subsequent short‐ and long‐term neurodevelopmental outcomes for the child. Regional alterations in mean brain T2* values should also be explored in future studies as motion correction, spatial resolution and alignment post processing techniques improve.

## 6 CONCLUSION

This study provides further evidence that alterations in brain development occur prior to preterm delivery. In the future, this may provide an opportunity to administer neuroprotective agents or alter the delivery timing to attenuate the significant neurodevelopment sequelae associated with sPTB.

## CONFLICT OF INTEREST STATEMENT

The authors have no conflicts of interest to declare.

## Data Availability

Data are available on request to academic institutions.
